# *In Silico* Phylogenetic and Structural Analyses of Plant Endogenous Danger Signaling Molecules upon Stress

**DOI:** 10.1155/2019/8683054

**Published:** 2019-07-15

**Authors:** Athanasia Pavlopoulou, Ezgi Karaca, Alma Balestrazzi, Alexandros G. Georgakilas

**Affiliations:** ^1^Izmir International Biomedicine and Genome Institute, Dokuz Eylül University, 35340 Balcova, Izmir, Turkey; ^2^Izmir Biomedicine and Genome Center, 35340 Balcova, Izmir, Turkey; ^3^Department of Biology and Biotechnology “Lazzaro Spallanzani”, University of Pavia, Via Ferrata 1, 27100 Pavia, Italy; ^4^DNA Damage Laboratory, Department of Physics, School of Applied Mathematical and Physical Sciences, National Technical University of Athens (NTUA), Athens, Greece

## Abstract

The plant innate immune system has two major branches, the pathogen-triggered immunity and the effector-triggered immunity (ETI). The effectors are molecules released by plant attackers to evade host immunity. In addition to the foreign intruders, plants possess endogenous instigators produced in response to general cellular injury termed as damage-associated molecular patterns (DAMPs). In plants, DAMPs or alarmins are released by damaged, stressed, or dying cells following abiotic stress such as radiation, oxidative and drought stresses. In turn, a cascade of downstream signaling events is initiated leading to the upregulation of defense or response-related genes. In the present study, we have investigated more thoroughly the conservation status of the molecular mechanisms implicated in the danger signaling primarily in plants. Towards this direction, we have performed *in silico* phylogenetic and structural analyses of the associated biomolecules in taxonomically diverse plant species. On the basis of our results, the defense mechanisms appear to be largely conserved within the plant kingdom. Of note, the sequence and/or function of several components of these mechanisms was found to be conserved in animals, as well. At the same time, the molecules involved in plant defense were found to form a dense protein-protein interaction (PPi) network, suggesting a crosstalk between the various defense mechanisms to a variety of stresses, like oxidative stress.

## 1. Introduction

Plants lack a global immune surveillance system, but they have acquired through evolution a highly effective innate immune response. There are currently three major types of elicitors of immune response in plants: (a) the “nonself” foreign molecule microbial/pathogen-associated molecular patterns (MAMPs/PAMPs) recognized by surface-localized pattern recognition receptors (PRRs), (b) signals produced by herbivores, nematodes, or parasitic plants, and (c) their effectors released by the attackers in order to counteract host defense [1]. Based on the principle on the wide variety of all these patterns inducing immune response, a unifying converging theory has emerged that points towards the concept of host perception of “danger” independently of its origin [2, 3].

DAMPs as death-, danger-, or damage-associated molecular patterns are of biological origin and are considered the major immunogenic mediators released passively by damaged, stressed, or dying cells, including tumor cells targeted by oxidative injury, radiation, or chemotherapy [4]. Endogenous or self signals are of two kinds, the primary DAMPS (e.g., cell wall fragments) and the secondary signals produced in response to danger, like processed protein fragments, such as phytocytokines, similar to animal cytokines. A third class that has evolved lately and is not well-defined includes the abiotic danger signals, like nanomaterials [5]. DAMPs are host cell-derived, as opposed to exogenously derived (nonself) MAMPs and PAMPs [6]. In a seminal study on this field by Matzinger [7], it was suggested that the immune system does not actually distinguish between self and nonself but rather detects “danger” through a series of positive and negative signals derived from damaged or stressed tissues mediated by DAMPs [7]. In plants, DAMPs, similar to animals, are perceived by pattern recognition receptors (PRRs), such as plasma membrane-localized receptors, thereby leading to a cascade of events including cytoplasmic Ca^2+^ elevation, depolarization of the cell membrane, production of reactive oxygen species (ROS), the transient phosphorylation of mitogen-activated protein kinases (MAPKs), and the transcriptional upregulation of defense or response-related genes [6, 8, 9]. In humans, oxidative stress, through the production of ROS, activates components of the MAPK-mediated signaling pathway including ERK, JNK, and p38 MAPKs; this can have both prosurvival and proapoptotic effects [10]. Although in yeast and mammals the main mechanisms triggering MAPK signaling activation under stress conditions have been studied extensively, in plants they remain largely unexplored [11].

In plant cells, there is emerging evidence of the connection between DAMPs and the DNA damage response (DDR), as nucleic acid recognition represents a fundamental step in host defense. Plants have been documented to perceive both extracellular DNA and RNA [12–14]. Toll-like receptors (TLRs) play a central role in the preferential recognition and binding of extracellular DNA in animals [15–19]. Despite the fact that TLR homologs have not been identified in plants, extracellular self-DNA (sDNA) was shown to act as a DAMP in plants. It has been reported that sDNA can trigger ROS- and MAPK-dependent signaling cascades [20], alter the CpG DNA methylation status (hypomethylation), and elicit defense-related responses [21]. Moreover, DNA damage and alteration of the primary chromatin structure were shown to induce the expression of defense-related genes [22, 23]. Elucidation of the plant-specific receptors that recognize extracellular sDNA as DAMPs could advance our knowledge on sDNA-dependent danger signaling in plant cells.

Plants have evolved mechanisms of innate immunity against detrimental pathogenic microorganisms and herbivorous animals. As mentioned above, DAMPs or alarmins, i.e., biomolecules released by stressed cells, share similarities between plants and animals in different aspects. For example, the recently discovered *Arabidopsis* HMGB3 is the counterpart of the pivotal animal DAMP HMGB1 [6]. Recent studies highlight the role of plants as unique biological models to monitor various types of exogenous (environmental) stress. More specifically, green leaf volatiles (GLVs) can act as airborne infochemicals that regulate the expression of defense response-related plant genes as shown in *Arabidopsis thaliana* [24]. In addition, medicinal plants and their derivatives can mitigate nephrotoxicity and anticancer drug side effects due to their intrinsic antioxidant and anti-inflammatory properties (reviewed in [25]). It would be necessary for the better understanding of the complex response network of various organisms to abiotic stresses to highlight the key genes and the interactions of this network to inflammatory and immune response networks. DAMPs are regarded as the link between these networks. Therefore, the analytical description of the molecular mechanisms and regulatory pathways that orchestrate plant responses to abiotic stresses and, particularly, oxidative stress, cell death, and DDR is crucial for the application of this knowledge to more complicated organisms.

Plants' immune response against intruders is regulated mainly by two antagonistic defense signaling pathways: (i) the salicylic acid- (SA-) mediated signal transduction pathway elicited by biotrophic and hemiobiotrophic pathogens and (ii) the octadecanoid signaling pathway with the key hormone jasmonic acid (JA) induced by heterotrophic pathogens and herbivores [1]. Of note, the oxidized lipids that participate in octadecanoid signaling are the plant functional equivalents of the mammalian oxidized phospholipids [26].

To date, there is still a knowledge gap concerning the evolutionary origin of the different DAMP-dependent signaling cascades identified in the plant kingdom and the way this valuable knowledge can be projected to more complex organisms, especially animal cells. We believe that an evolutionary perspective could provide useful insights into the origin of DAMP-mediated mechanisms and their role across the different levels of biological organization and complexity. In this study, we have made an effort to assess the conservation of the defense mechanisms by conducting phylogenetic and structural analyses of the molecules implicated in different stages of danger signaling in 11 vascular plant species that represent diverse taxonomic divisions: *Arabidopsis thaliana* (thale cress), *Zea mays*, *Oryza sativa* (rice), *Hordeum vulgare* (barley), *Medicago truncatula* (barrel medic), *Nicotiana tabacum* (tobacco), *Populus trichocarpa* (cottonwood), *Solanum lycopersicum* (tomato), *Prunus persica* (peach), *Vitis vinifera* (wine grape), and *Pisum sativum* (pea).

## 2. Methods

### 2.1. Sequence Dataset and Homology Searching

The bibliographic database PubMed/MEDLINE (https://www.ncbi.nlm.nih.gov/pubmed) was thoroughly searched using relevant keywords such as “damage-associated molecular patterns”, “danger signals”, “alarmins”, “danger-associated molecular patterns”, “DAMPs”, “endogenous danger signaling”, “plants”, and “viridiplantae”. The names and/or accession numbers of the characterized proteins reported in the articles were used to retrieve their corresponding sequences from the publicly accessible sequence databases UniProtKB [27] and NCBI's GenBank [28]. To identify more orthologous protein sequences, the known sequences were used as a query in reciprocal BLASTp and tBLASTn [29] searches (cutoff *E* − value ≤ 1.0*E* − 9) of the genomes of 11 vascular plant species, representing diverse taxonomic divisions (Figure S1). The canonical or longest transcripts were selected. Any partial or ambiguous sequences were not included in the subsequent steps of the study.

### 2.2. Phylogenetic Analyses

The amino acid sequences of the corresponding proteins (Table S1) were aligned with MAFFT, version 3.7 [30]. Phylogenetic analyses were performed by employing a maximum likelihood (ML) method. To this end, the resulting multiple alignments were provided as input to PhyML v.3.0 [31], which optimizes a distance-based starting tree (BioNJ) [32] by employing a heuristic algorithm. The expected number of amino acid substitutions per site was estimated with the JTT model [33]. Protein sequences distantly related to those under study were used as outgroups. The robustness of the reconstructed phylogenetic trees was evaluated by bootstrapping (200 bootstrap pseudoreplicates). Trees were illustrated using Dendroscope [34].

### 2.3. Pairwise Distance Estimation

Pairwise distances between protein sequences were computed using the software package MEGA, version 7.0 [35].

### 2.4. Protein Domain Organization

The protein sequences under study were searched against the protein signature databases SMART v.8.0 [36] and CDD v.3.16 [37] in order to determine the boundaries of their constituent domains.

### 2.5. Sequence Motifs

The amino acid sequences corresponding to the catalytic kinase domain of the orthologous proteins DORN1, MAPK3, MAPK6, PEPR1/2, SERK3/4/5, SR160, and WAKL (Table S1) were aligned using MAFFT v.3.7 [30] and edited with Utopia's CINEMA alignment editor [38]. Ungapped sequence motifs were extracted from the alignment and submitted to WebLogo 3.5.0 [39] with default parameters, to create consensus sequences.

### 2.6. Tertiary Structure Analysis

The plant MAPK3 and MAPK6 sequences under study were aligned using the PROMALS3D multiple sequence alignment program, which incorporates evolutionary and tertiary structural information to improve alignment accuracy [40, 41]. The degree of conservation of amino acid residues of the homologous plant MAPK3 and MAPK6 proteins was estimated with the usage of the ConSurf program [42]. To this end, the resulting multiple sequence alignment of the MAPK3 and MAPK6 amino acid sequences was provided as input to ConSurf to map the conservation grades of the amino acid residues onto the resolved tertiary structure of the *Arabidopsis thaliana* MAPK6 (PDB ID: 5CI6 [43]). The protein structure was displayed using the PyMOL molecular graphics program (http://www.pymol.org/).

### 2.7. Structural Modeling of Protein-Protein Interactions

The tertiary structure of the extracellular LRR domain of the *A. thaliana* PEPR2 was predicted by homology modeling. The X-ray crystal structure of the *Arabidopsis* PEPR1LRR (PDB ID: 5GR8, chain A [44]) was used as a template to model PEPR2LRR using the I-TASSER server [45]. The quality of the final modeled protein structures was evaluated with Procheck [46].

In order to assemble the trimeric PEPR2LRR-AtPEP1-BAK1 complex, the following steps were carried out: LRR domains (italicized) present in the *PEPR1LRR*-AtPEP1 (PDB ID: 5GR8) and *FLS2LRR*-FLG22-BAK1 (PDB ID: 4MN8) complexes and the *PEPR2LRR* homology model were structurally superimposed by using the alignment algorithm FATCAT [47]. Following this superimposition, the coordinates of FLS2LRR-FLG22-BAK1 and PEPR1LRR were deleted. As a result of this procedure, a crude model of the PEPR2LRR-AtPEP1-BAK1 complex was obtained. This crude model was refined with the water refinement step of the HADDOCK webserver [48]. During refinement, the interaction between PEPR2LRR and AtPEP1 was used as a restraint, with the following restraint definition: (i) assign (resi 438 and segid A) (resi 23 and segid C) 3.00 3.00 0.05 and (ii) assign (resi 392 and segid A) (resi 23 and segid C) 3.00 3.00 0.05. All of the interface statistics were carried out through the CoCoMaps tool (https://www.molnac.unisa.it/BioTools/cocomaps/).

### 2.8. Functional Association Network

The associations among the plant proteins implicated in danger signaling, in this study, were investigated using STRING v11 [49], a database of either experimental or predicted, direct or indirect, protein-protein interactions; a relatively high confidence interaction score (0.7) was selected. Intermediate nodes connecting the input nodes were also predicted, with a maximum number of 5 interactors. To this end, the *Arabidopsis thaliana* proteins WAK1, MAPK3, MAPK6, DORN1, PEPR1, PEPR2, SR160, HMGB1, HMGB2, HMGB3, SERK3, and SERK4 were used as input to STRING to generate an interaction network.

## 3. Results and Discussion

### 3.1. Classes of DAMPs

The diverse DAMPs that have been identified in plants are arbitrarily grouped, in this study, in the following classes.

#### 3.1.1. Cell Wall-Derived DAMPs

Oligogalacturonide (OG) fragments are released from the degradation of the plant cell wall constituent homogalacturonan (HGA) by pathogen-encoded polygalacturonases. OG fragments trigger a danger signaling cascade involving ROS production, increase in cytoplasmic Ca^2+^ concentration, activation of MAPK3 and MAPK6, upregulation of resistance genes, and activation of components of the SA, JA, and ethylene pathways [50–52]. The wall-associated kinase 1 (WAK1) was suggested to perceive OGs [53, 54]. Besides OGs, Claverie et al. suggest that xyloglucans (Xh), components of the cell wall hemicellulose, act as DAMPs and induce defense responses similar to OGs [55].

Likewise, in animals, glycosaminoglycan hyaluronan fragments are released during damage and are implicated in wound repair and regeneration. These fragments are recognized by the Toll-like receptors TLR2 and TLR4, leading to activation of the inflammatory gene expression [56]. Of note, hyaluronan fragments and MAPK activation were found to mediate ROS-induced upregulation of the defense-related gene MUC5AC [57].

**Figure 1:**
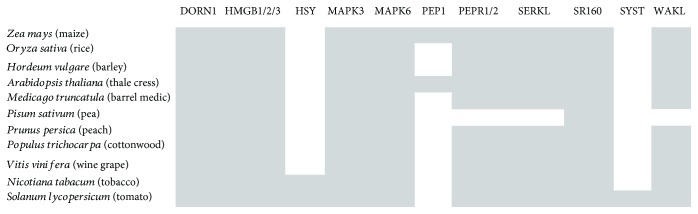
Phylogenetic distribution of the proteins implicated in plant defense.

**Figure 2:**
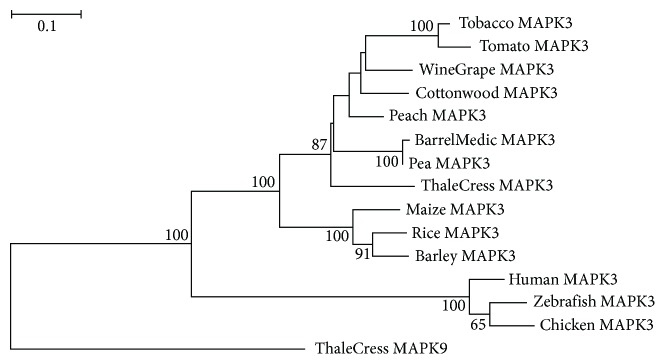
ML-based tree of MAPK3 protein sequences. The branch lengths are proportional to the evolutionary distance. Bootstrap support values ≥ 50% are shown at the nodes. The scale bar at the upper left denotes the length of amino acid replacements per position. The sequence ThaleCress MAPK9 was used as outgroup.

**Figure 3:**
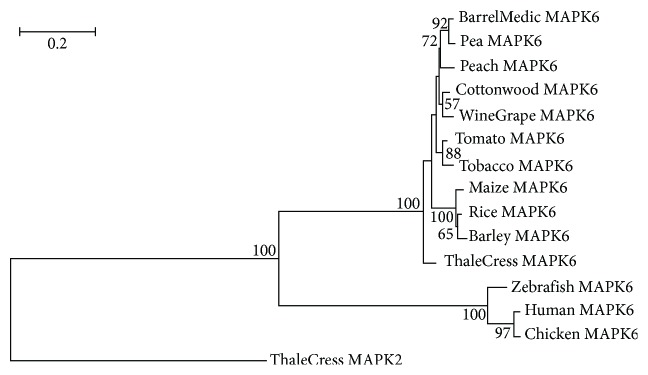
Tree of MAPK6 protein sequences. The sequence ThaleCress MAPK9 was used as outgroup. The conventions are the same as in Figure 2.

The viridiplantae MAPK3 and MAPK6 proteins (Figure 1) have orthologs in metazoa which are also implicated in danger signaling [58]. The plant MAPK3 and MAPK6 have rather evolved independently from their animal orthologs as they form distinct monophyletic groups with bootstrap values of 100, and they are connected to the corresponding metazoan clade with a long branch which reflects long evolutionary distance (Figures 2 and 3). The monocotyledonous plant MAPK3 and MAPK6 sequences form well-separated clades in their respective phylogenetic trees, leading to the suggestion that these sequences have probably appeared after the monocot-eudicot divergence about 200 million years ago (MYA) [59]. The tomato and tobacco MAPK3 and MAPK6 sequences cluster together with high statistical confidence, indicating that these sequences emerged after the members of the order solanales branched off from the other vascular plants (Figures 2 and 3).

**Figure 4:**
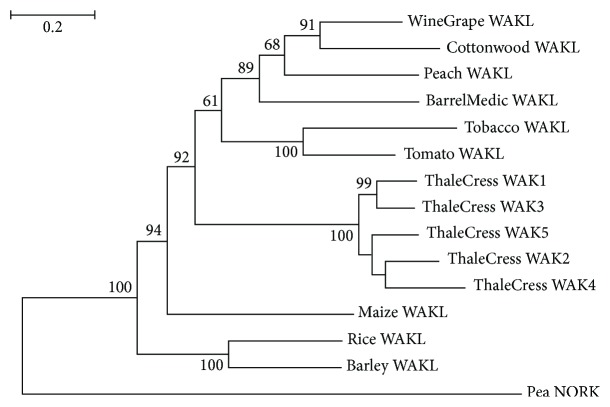
Tree of WAKL protein sequences. The sequence Pea NORK (nodulation receptor kinase) was used as outgroup. The conventions are the same as in Figure 2.

A cluster of five highly similar *WAK (WAK1-5)* genes are present in *A. thaliana* (thale cress) [60]. The *Arabidopsis* WAK1-5 protein sequences form a separate highly supported clade (Figure 4). Based on sequence database searches, a single copy of *WAK* was found in the other plant species (Figure 1). *WAK* homologs were not detected in the pea; this is probably due to incomplete annotation of the *Pisum sativum* genome. The encoded protein was arbitrarily referred to as WAKL, where “L” stands for “like,” by virtue of sequence similarity to the thale cress WAK1-5 (Figure 4). A primordial *WAK* gene might have undergone a series of duplications in *A. thaliana*, thereby giving rise to fıve *WAK* paralogs. However, any fully sequenced and annotated genomes of other members of the order brassicales are not currently available, apart from the one of the model plant organism *A. thaliana*, which would allow us to examine whether the *WAK* gene expansion is species- or order-specific. Based on extensive bibliographic searches, no experimental evidence was found about *A. thaliana* WAK2-5 acting as PRRs. WAK1 has apparently acquired a more specialized function during the course of evolution. WAK proteins are likely restricted to vascular plants, since orthologous WAK sequences were not found in metazoa. The role of these WAKL proteins in the perception of OGs in their host plant organisms remains to be investigated experimentally.

#### 3.1.2. Extracellular ATP

Extracellular ATP (eATP), released from stressed or damaged cells into the extracellular milieu, constitutes a class of danger signals both in plants and animals. In plants, eATP plays an important signaling role in plant cells by participating in several processes including cell development, viability, and stress responses [61, 62]. It is recognized by the plant-specific PRR, DORN1 (DOes not Respond to Nucleotides 1), which is a membrane lectin receptor kinase [63]. DORN1 is required for the ATP-mediated intracellular influx of Ca^2+^, formation of ROS, phosphorylation of MAPK3 and MAPK6, and stimulation of the expression of defense genes [64, 65]. Tripathi and colleagues demonstrated that eATP elicits defense responses via the JA signaling pathway [66].

**Figure 5:**
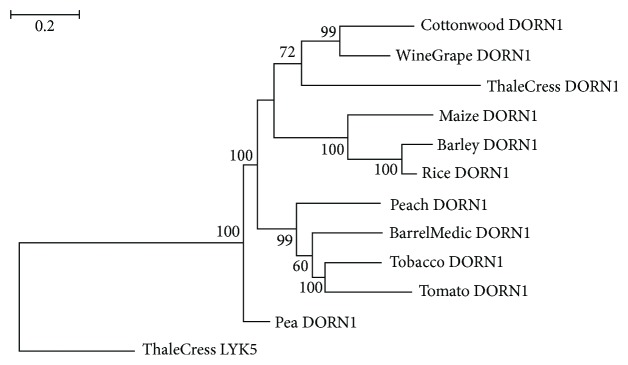
Tree of DORN1 protein sequences. The sequence ThaleCress LYK5 was used as outgroup. The conventions are the same as in Figure 2.

Putative DORN1 homologous sequences were detected in all plant species under investigation (Figure 1). The cereal plants as well as the solanales DORN1 sequences form their own monophyletic group with bootstrap values of 100, suggesting class- and order-specific evolution of *DORN1* (Figure 5). DORN homologous sequences were found exclusively in plants, wherein might be implicated in the recognition of eATP.

In animals, eATP was shown to mediate oxidative stress response [67, 68] and to be involved in inflammatory responses [64, 69]. Two types of P2 purinoceptors, the ligand-gated cation channel P2X receptors and the metabotropic (G-protein-coupled) P2Y receptors [70], are activated by eATP and are involved in the eATP-induced increase of cytosolic Ca^2+^ levels [71].

#### 3.1.3. Polypeptide-Based DAMPs

A wide range of polypeptide-based DAMPs derived from larger precursors can act as robust inducers of plant defense responses [72, 73]. In *Arabidopsis thaliana*, the 23-amino acid PEP1 (Plant Elicitor Peptide) is processed from a 92-amino acid precursor upon damage, JA, and ethylene [74]. Based on homology searches, only one ortholog of AtPEP1 was detected in the fellow plants, that is, ZmPEP1, in maize. This protein is considered a DAMP, as well, given that it acts as an endogenous elicitor able to regulate the pathogenesis-related gene through sucrose-mediated signaling [75]. In *A. thaliana*, PEP1 is perceived by two PRRs, PEPR1 (PEPtide Receptor 1) and PEPR2, leading to ROS production and upregulation of defensin-like genes [76]. Of note, the only PEP orthologs were found in two species belonging to divergent taxonomic groups, eudicots (*A. thaliana*) and monocots (*Zea mays*), and not any taxonomically related species (such as fellow eudicots). This suggests that even distantly related organisms respond to similar stimuli with similar mechanisms.

**Figure 6:**
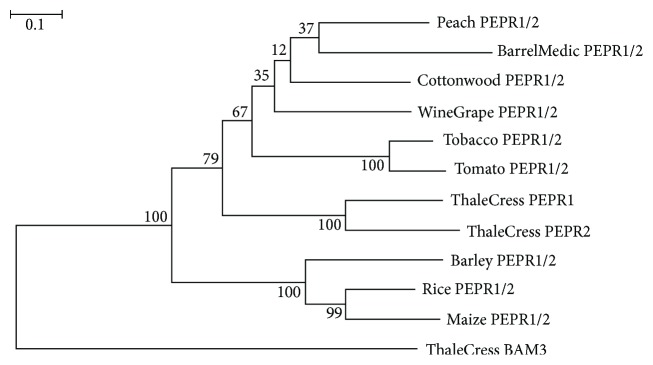
Tree of PEPR1/2 protein sequences. The sequence ThaleCress BAM3 was used as outgroup. The conventions are the same as in Figure 2.

PEPs' cognate receptors, however, were found in all plants under study (except pea) (Figure 1). Thale cress harbors a total of seven PEPs (PEP1-PEP7) and two *PEPR* paralogs, the encoded proteins of which share 67% identity. In the inferred phylogenetic tree, the AtPEPR1 and AtPEPR2 protein sequences form a highly monophyletic branch with bootstrap support 100 (Figure 6). However, a single copy of *PEPR* was detected in the other plant species; the corresponding protein of which is arbitrarily called PEPR1/2 due to its amino acid sequence similarity to both AtPEPR1 and AtPEPR2. The cereal plants form a separate highly supported clade, leading to the suggestion that *PEPR1/2* genes evolved after the monocots-dicots split (Figure 6). *PEPRs* are also kingdom-restricted, since *PEPR* orthologs were detected exclusively in vascular plants.

AtPEPs exhibit functional similarity to the 18-amino acid peptide systemin, which is processed from the 200-amino acid hormone prosystemin (SYST) in the tomato upon wounding [77]. Prosystemin was found only in the tomato and not in tobacco which is also member of the same solanales order (Figure 1). However, two hydroxyproline-rich systemin (HSY) peptides, designated HSYA and HSYB, cleaved from a larger preprotein, were identified in tobacco (Figure 1) which do not bear any sequence similarity to systemin [77]. Both SYST and HSY induce a JA-mediated signaling pathway that leads to the activation of defense-related genes [77, 78].

Of particular note, as in the case of AtPEPRs, systemin cognate receptors (SR160) were detected in all plant species under investigation (Figure 1). It would be intriguing to suggest that *PEPR* and *SR160* genes were probably propagated through vertical gene transfer in plants, and the peptides that specifically bind to them evolved later in certain species in order to carry out species-specific functions.

Protein fragments, such as fibronectin fragments (FN-fs), are the equivalents of the plant polypeptide-based DAMPs in mammals, and they are perceived by Toll-like receptors (TLRs), like TLR4 [79]. Of note, FN-fs were found to stimulate ROS production in human articular chondrocytes [80].

PEPR1/2 and SR160 are transmembrane (TM) proteins comprised of LRR (Leucine-Rich Repeats) motifs, one TM domain, and one catalytic kinase domain in the cytoplasmic region. PEPRs resemble the mammalian TLRs in terms that they share LRR motifs in their amino-termini, suggesting that the extracellular LRR domain is used for DAMP perception both in plants and mammals [76, 81].

**Figure 7:**
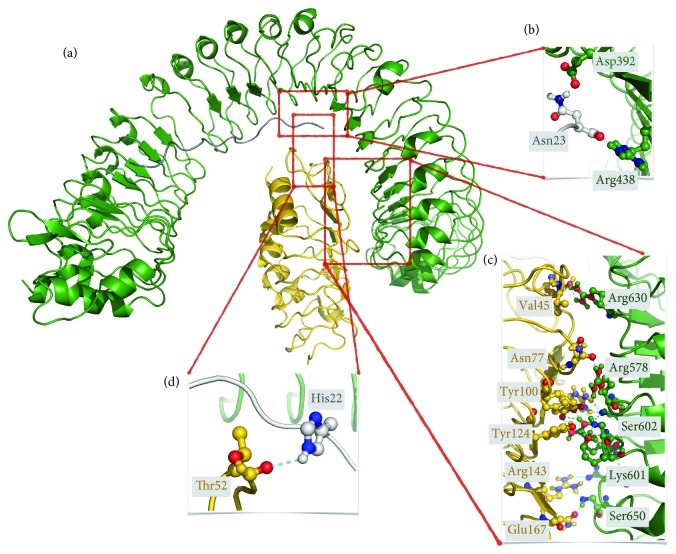
(a) Structural model of the *Arabidopsis thaliana* PEPR2LRR (green)-AtPEP1 (gray)-BAK1 (gold) complex. Important polar interactions formed across the PEPR2LRR and AtPEP1, PEPR2LRR and BAK1, AtPEP1 and BAK1 interfaces are depicted in (b), (c), and (d), respectively. Polar interactors are represented in ball-and-sticks, with oxygen atoms colored in red and nitrogens in blue.

In this study, we aimed to get an evolutionary glimpse into the binding pattern of the *A. thaliana* PEPR2LRR to AtPEP1. The interaction between PEPR2LRR-AtPEP1 (Figure 7) was also modeled by using the crystal structure of PEPR1LRR-AtPEP1 (PDB ID: 5GR8) as a template. According to 5GR8, there are 16 hydrogen bonds formed between PEPR1LRR and its AtPEP1 peptide [44]. In our PEPR2LRR-AtPEP1 model, this number increases to 17. Two hydrogen bonds, among all, were found to be strictly conserved, i.e., the ones between TYR395-GLN21 and ASP441-ASN23 across the PEPR1LRR-AtPEP1 interface and TYR346-GLN21 and ASP392-ASN23 across the PEPR2LRR-AtPEP1 interface. This reflects the importance of these stabilizing interactions within the PEPR family. The extent of the PEPR1LRR-AtPEP1 and PEPR2LRR-AtPEP1 interfaces appear to be conserved, where 66 amino acids interact over an area of 1033 Å^2^ in the case of PEPR1LRR-AtPEP1, and 65 amino acids interact through a 1046 Å^2^-sized interface. PEPR2LRR-BAK1 and its template FLS2LRR-BAK1 (PDB ID: 4MN8) entail a similar number of amino acids across their interfaces (57 for PEPR2LRR-BAK1 and 56 for FLS2LRR-BAK1) (Figure 7). Although this is the case, PEPR2LRR-BAK1 makes more polar interactions (through 12 hydrogen bonds) compared to FLS2LRR-BAK1 (entails 5 hydrogen bonds only), indicating the polar character of the PEPR2LRR surface and a possibly higher affinity between PEPR2LRR-BAK1. Of particular interest, in this study, the structurally important residues TYR395 and ASP441 in AtPEPR2LRR were also found to be conserved in the fellow plant PEPR1/2 primary amino acid sequences, based on multiple sequence alignment. This leads to the suggestion that the *Zea mays* PEPR1/2LRR could possibly bind to its corresponding ZmPEP1.

#### 3.1.4. HMGB3

A relatively novel class of plant DAMPs, the *Arabidopsis thaliana* HMGB3 (High-Mobility Group Box 3), was shown to induce plant innate immunity, including activation of MAPK3/4/6 and defense-related genes, as well as increased resistance to necrotrophic pathogens. This response is mediated by the LRR-PRR BAK1 (Brassinosteroid insensitive 1-Associated Kinase 1)/SERK3 (Somatic Embryogenesis Receptor-like Kinase 3) and BKK1 (BAK1-like 1)/SERK4 [82].

The mammalian counterpart of the plant HMGB3, HMGB1, is released upon tissue wounding and inflammation [83], as well as response to oxidative [84] and ionizing radiation-induced stress [85]. Release of HMGB1 into the extracellular milieu is associated with the occurrence of severe stress that may have a negative effect on tissue function and homeostasis [85]. HMGB1 is a chromosomal scaffold protein involved in chromatin remodeling induced in response to DNA damage accumulation. It promotes nucleotide excision repair (NER), base excision repair (BER), and mismatch repair (MMR) pathways, facilitating DNA protein kinase (DNA-PK) activity [86]; similarly to the catalytic subunit of DNA-PK, DNA-PKcs, HMGB1 binds CpG oligonucleotides, inducing the TLR9-mediated inflammatory response [87]. Based on protein domain analysis, the human HMGB1 harbors two HMG boxes, BoxA and BoxB, whereas the *A. thaliana* HMGB proteins have only one HMG box. In the plant species under study, single HMGB homologs were detected and were found to be related to *Arabidopsis* HMGB1/2/3. This is because the *A. thaliana* proteins HMGB1, HMGB2, and HMGB3 share a high degree of sequence identity/similarity. HMGB1/2/3-related sequences were detected in all plant species (Figure S1). This finding might represent an interesting starting point to explore more in depth the correlation between DAMPs and DDR also in plants and to provide clues about the degree of conservation, at the *trans*-kingdom level, of such molecular processes.

Genes encoding SERKs have been identified in the genomes of vascular plants, both monocots and dicots [88]. Furthermore, Sasaki et al. [89] reported the presence of SERK homologs in the less evolved plant liverwort *Marchantia polymorpha* and even in the unicellular green alga *Closterium ehrenbergii*. *SERKs* are ancient, essential genes, conserved during speciation. Multiple *SERK* genes are found in dicots, monocots, and nonvascular plants. This indicates that SERK genes are at least 450 million years old and were present before the split of nonvascular and vascular plants [90]. Putative SERK-related proteins were detected in the species under investigation (except pea) (Figure 1).

### 3.2. Conserved Structural Features of Plant Proteins Implicated in Danger Signaling

**Figure 8:**
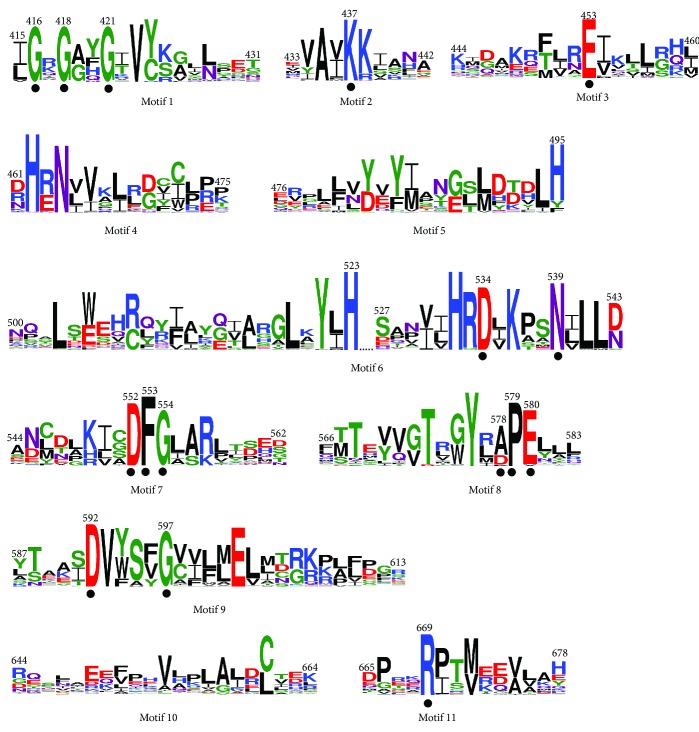
Sequence logos of the conserved protein motifs identified in the catalytic domain of protein kinases. The first and last amino acid residues of each motif are numbered according to *A. thaliana* WAK1. The conserved/invariant amino acid residues critical for the protein kinase activity are denoted by dots. The height of each letter depicts the relative frequency of the corresponding amino at that position, and the letters are ordered in such a way that the most frequent one is on the top.

In the present study, the 11 conserved catalytic sequence motifs reported by Hanks et al. [91] were identified in the kinase domain of the plant proteins DORN1, MAPK3, MAPK6, PEPR1/2, SERK3/4/5, SR160, and WAKL investigated in this study (Figure 8). In motif 1, the consensus signature GxGxxG of phosphate binding, conserved across nucleotide binding proteins [92, 93], was detected. The invariant lysine (K) in motif 2 is essential in protein kinase activity as it participates actively in the phosphate transfer reaction [92]. The invariant aspartate (D) and asparagine (N) amino acids in motif 6, as well as the stretch of [94] Asp (D), Phe (F), and Gly (G) residues in motif 7, are actively involved in ATP binding. In motif 8, the highly conserved Glu (E) and invariant Pro (P) were found to be flanked by a conserved alanine (A) residue in [91]. However, in the plant kinase domain, at the same position, Asp (D) was found to be the most frequent residue instead. Phosphorylation of these residues was associated with enhanced catalytic activity in a number of protein kinases [95, 96]. Moreover, several highly conserved amino acids with an unknown role identified by Hanks et al. [91] were also found to be conserved in plant kinases, including Asp (D) in motif 3, Asp (D) and Gly (G) in motif 9, and Arg (R) in motif 11.

**Figure 9:**
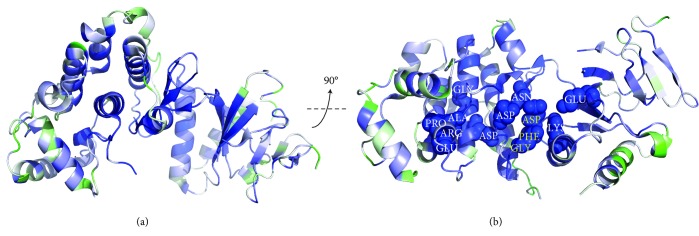
(a) Tertiary structure of the *Arabidopsis thaliana* MAPK6 (PDB ID: 5CI6), where blue corresponds to the most highly conserved region and green to the least one. The conserved DFG (207-209) motif is highlighted in yellow ((b), at the center of the molecule). The other important conserved residues can be listed as (from left to right) GLY251, ALA 231, PRO232, GLU233, ARG336, ASP246, ASP189, ASN194, GLU110, and LYS92. The numbering is given as in 5CI6.

The degree of conservation of the amino acid residues critical for kinase activity is depicted in the three-dimensional structure of *A. thaliana* MAPK6 [43] (Figure 9). A total 13 out of the 16 amino acids found to be conserved/invariant in the primary structures of the kinase domain of plant proteins (Figure 8) are also shown to be conserved in the tertiary structure of this domain (Figure 9).

### 3.3. Functional Interactome

**Figure 10:**
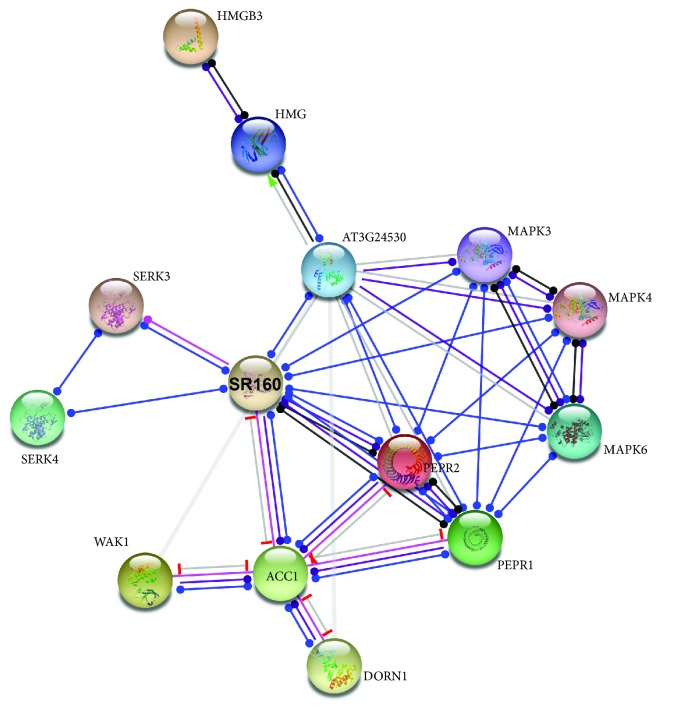
Functional network of the *Arabidopsis thaliana* molecules involved in DAMP-mediated danger signaling. The nodes represent proteins, and the lines denote edges.

The input *A. thaliana* molecules involved in plant defense appear to form a dense network (Figure 10), leading to the suggestion that the individual pathways (e.g., DORN1-mediated pathway, etc.) interact into a rather complex plant endogenous danger signal transduction network. For example, the DAMP receptors DORN1, WAK1, PEPR1/2, and SR160 appear to be connected through the multifunctional enzyme ACC1 (acetyl-CoA carboxylase 1), while SERK3/4 interacts directly with SR160. The ubiquitous acetyl-CoA carboxylase is a pivotal enzyme in the synthesis of fatty acids in both eukaryotes and prokaryotes. Both plastid and cytosolic ACC1/2 are nuclear-encoded multidomain enzymes of eukaryotic origin and have central roles in the control of cell division and differentiation and most importantly in many biosynthetic and homeostatic mechanisms [97]. ACC1 catalyzes the key reaction in the biosynthesis of very-long-chain fatty acids giving rise to cuticular waxes, suberin, and sphingolipids, and it is connected to several abiotic and biotic stress response networks [98]. Of note, cuticular waxes play active roles in both local and systemic resistance during plant-pathogen interaction since the plant cuticle is part of the first step of defense pattern-triggered immunity that includes DAMPs [99]. The predicted interaction between ACC1 and the several DAMP receptors might reflect some aspects, still uncovered, of the mechanisms underlying the plant resistance or susceptibility to pathogens. It has been suggested that decreased levels of specific cutin monomers or wax components contribute to the decreased expression of virulence factors or may act as receptors triggering the defense-associated signaling pathway [100]. Further investigation towards elucidating the functional or physical associations between the master regulator of fatty acids ACC1 and the DAMP receptors would provide us with a more detailed picture of the ACC1-DAMP interactome in plants.

## 4. Conclusions

It has been only about two decades when the significance of DAMPs for the survival and homeostasis of multicellular organisms under stress conditions has been highlighted. A wide range of molecules have been recognized as DAMPs, and related patterns have been deciphered, whereas a DAMP-like behavior has been evidenced for other molecules. According to Rubartelli [101], the acute phase protein serum amyloid A (SAA) can be considered as a DAMP-like molecule because it is able to prime glial cells and activate the inflammasome in the absence of a pathogen-derived priming stimulus. This response is observed following brain injury and allows to induce the inflammatory response under completely sterile conditions [101]. In plants, a DAMP-like behavior has been described for methanol, a volatile byproduct of pectin methylesterases, rapidly released from the plant cell wall in response to pathogens [102]. Differently from other DAMPs, methanol has no elicitor activity; however, its solvent properties could affect the cell wall and plasma membrane-bound proteins and plasma membrane integrity, resulting in damaged-self signal [102]. These findings further support the apparent complexity of the DAMPs-related processes and functions. Related to more than twenty DAMPs discovered in animals to date, relatively few have been recognized in plants [6].

In our study, we have focused on the well-characterized plant DAMPs. We have performed comprehensive phylogenetics and protein structural analyses in order to identify “true” orthologs between and within the plant and animal kingdom. Our findings support the notion that all efforts to elucidate the pathway(s) through which innate immunity is triggered could likely uncover additional signaling components that are shared by all kingdoms, from plants to humans. The “ancestral” mechanisms of defense are conserved across kingdoms. Several key molecular “players” of those mechanisms appear to be largely conserved (e.g., eATP, calcium, and MAPKs). Some plant proteins involved in endogenous danger signaling were found to have sequence homologs in animals (e.g., MAPKs), whereas most of them were detected exclusively in plants. Both in plants and animals, selective pressure has been likely exerted to maintain the functional integrity of the defense mechanisms by recruiting or coopting into the existing molecular pathway novel players. These players may have no apparent sequence/structural similarity but, through convergent evolution, have rather acquired similar functions, as in the case of DORN1 and P2 purinoreceptors, in plants and animals, respectively.

These findings could provide the foundation for expanding the current knowledge on endogenous danger signaling molecules in plants and their role in adaptive defense mechanisms such as oxidative stress and other types of endogenous or exogenous stress. Of importance, based on the finding of this study, it would be tempting to suggest that plants could serve as basal model systems to study the individual signal transduction pathways implicated in danger signaling and further extrapolate this information to more complex organisms like mammals.

## Data Availability

The data used to support the findings of this study are available from the corresponding author upon request.

## Supplementary Materials

Supplementary Materials Figure S1: a cladogram extracted from NCBI's Taxonomy Database [103] depicting the evolutionary relationships among plant species. Table S1: accession numbers of the protein sequences investigated in the present study.
