# Supplementary material for: In Silico Phylogenetic and Structural Analyses of Plant Endogenous Danger Signaling Molecules upon Stress
Source: Oxid Med Cell Longev. 2019 Jul 15;2019:8683054. doi: 10.1155/2019/8683054 (PMC6668560; doi:10.1155/2019/8683054)
Supplement: Supplementary Materials — Figure S1: a cladogram extracted from NCBI's Taxonomy Database [103] depicting the evolutionary relationships among plant species. Table S1: accession numbers of the protein sequences investigated in the present study. [file 8683054.f1.pdf]

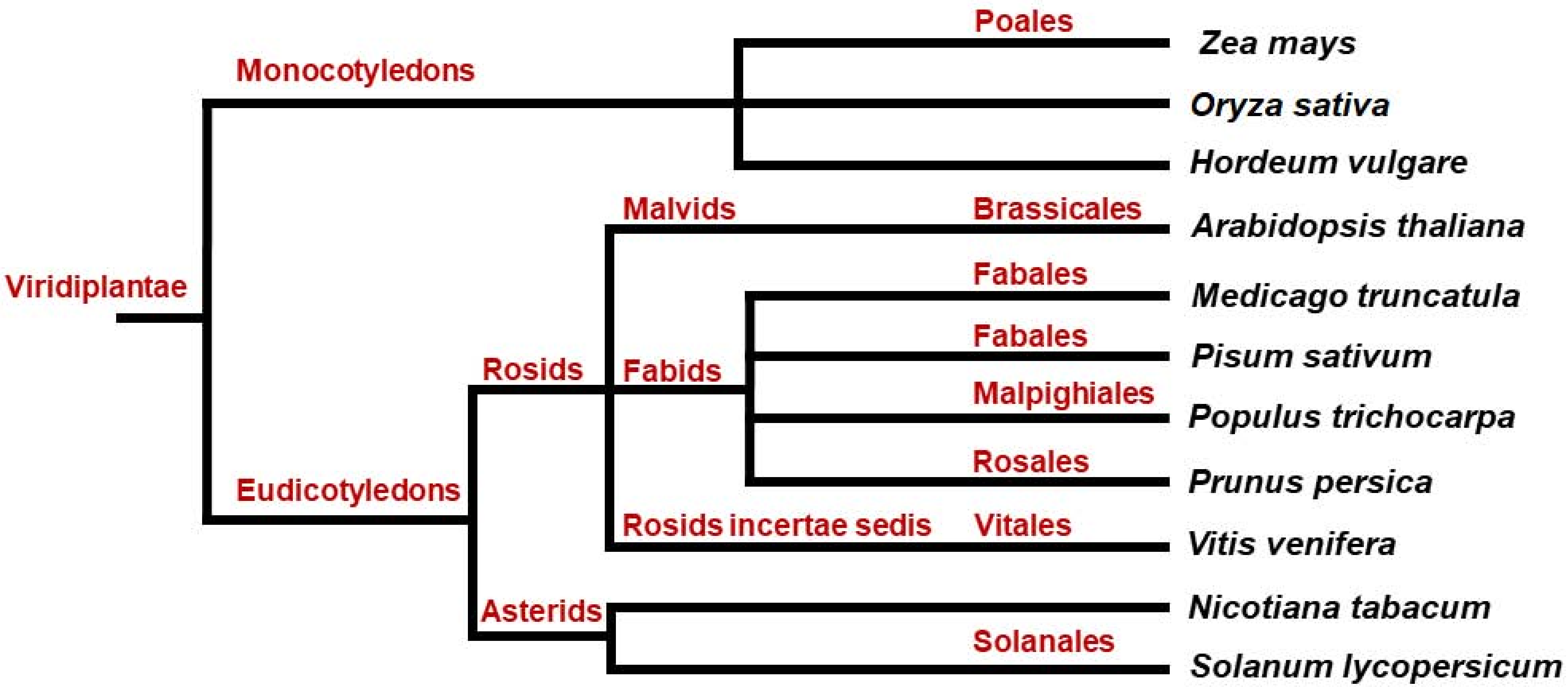
Poales
Zea mays
Monocotyledons
Oryza sativa
Hordeum vulgare
Malvids
Brassicales
Arabidopsis thaliana
Viridiplantae
Fabales
Medicago truncatula
Fabales
Pisum sativum
Rosids
Fabids
Malpighiales
Populus trichocarpa
Rosales
Prunus persica
Eudicotyledons
Rosids incertae sedis
Vitales
Vitis venifera
Asterids
Nicotiana tabacum
Solanales
Solanum lycopersicum

| Organism | Sequence | Accession code |
| --- | --- | --- |
| *Arabidopsis thaliana* | ThaleCress BAM3 | O65440[1] |
| *Arabidopsis thaliana* | ThaleCress DORN1 | Q9LSR8[1] |
| *Arabidopsis thaliana* | ThaleCress HMGB1 | O49595[1] |
| *Arabidopsis thaliana* | ThaleCress HMGB2 | O49596[1] |
| *Arabidopsis thaliana* | ThaleCress HMGB3 | P93047[1] |
| *Arabidopsis thaliana* | ThaleCress LYK5 | OAP11490[2] |
| *Arabidopsis thaliana* | ThaleCress MAPK2 | Q9S7U9[1] |
| *Arabidopsis thaliana* | ThaleCress MAPK3 | Q39023[1] |
| *Arabidopsis thaliana* | ThaleCress MAPK6 | Q39026[1] |
| *Arabidopsis thaliana* | ThaleCress MAPK9 | NP_974331[2] |
| *Arabidopsis thaliana* | ThaleCress PEP1 | Q9LV87[1] |
| *Arabidopsis thaliana* | ThaleCress PEPR1 | Q9SSL9[1] |
| *Arabidopsis thaliana* | ThaleCress PEPR2 | Q9FZ59[1] |
| *Arabidopsis thaliana* | ThaleCress SERK3 | Q94F62[1] |
| *Arabidopsis thaliana* | ThaleCress SERK4 | Q9SKG5[1] |
| *Arabidopsis thaliana* | ThaleCress SR160 | NP_195650[2] |
| *Arabidopsis thaliana* | ThaleCress WAK1 | Q39191[1] |
| *Arabidopsis thaliana* | ThaleCress WAK2 | Q9LMP1[1] |
| *Arabidopsis thaliana* | ThaleCress WAK3 | Q9LMN8[1] |
| *Arabidopsis thaliana* | ThaleCress WAK4 | Q9LMN6[1] |
| *Arabidopsis thaliana* | ThaleCress WAK5 | Q9LMN7[1] |
| *Medicago truncatula* | BarrelMedic DORN1 | XP_013448930[2] |
| *Medicago truncatula* | BarrelMedic HMGB1/2/3 | XP_013462296[2] |
| *Medicago truncatula* | BarrelMedic MAPK3 | XP_003606509[2] |
| *Medicago truncatula* | BarrelMedic MAPK6 | G7JNP9[1] |
| *Medicago truncatula* | BarrelMedic PEPR1/2 | XP_013465647[2] |
| *Medicago truncatula* | BarrelMedic SERKL | XP_003593155[2] |
| *Medicago truncatula* | BarrelMedic SR160 | XP_003602504[2] |
| *Medicago truncatula* | BarrelMedic WAKL | XP_013461333[2] |
| *Pisum sativum* | Pea DORN1 | ABU75307[2] |
| *Pisum sativum* | Pea HMGB1/2/3 | Q41026[1] |
| *Pisum sativum* | Pea MAPK3 | Q9M6S1[1] |
| *Pisum sativum* | Pea MAPK6 | Q06060[1] |
| *Pisum sativum* | Pea NORK | Q8LKZ1[1] |
| *Pisum sativum* | Pea SR160 | Q76FZ8[1] |
| *Prunus persica* | Peach DORN1 | XP_020422899[2] |
| *Prunus persica* | Peach HMGB1/2/3 | XP_007202727[2] |
| *Prunus persica* | Peach MAPK3 | XP_007205387[2] |
| *Prunus persica* | Peach MAPK6 | M5VPZ6[1] |
| *Prunus persica* | Peach PEPR1/2 | XP_007217286[2] |
| *Prunus persica* | Peach SERKL | XP_007201734[2] |
| *Prunus persica* | Peach SR160 | XP_007208382[2] |
| *Prunus persica* | Peach WAKL | ONI11223[2] |
| *Populus trichocarpa* | Cottonwood DORN1 | XP_006379022[2] |

| | | |
|---|---|---|
| *Populus trichocarpa* | Cottonwood HMGB1/2/3 | XP_002306980[2] |
| *Populus trichocarpa* | Cottonwood MAPK3 | XP_002314017[2] |
| *Populus trichocarpa* | Cottonwood MAPK6 | B9HGK0[1] |
| *Populus trichocarpa* | Cottonwood PEPR1/2 | XP_002311912[2] |
| *Populus trichocarpa* | Cottonwood SERKL | XP_006369340[2] |
| *Populus trichocarpa* | Cottonwood SR160 | XP_002310619[2] |
| *Populus trichocarpa* | Cottonwood WAKL | XP_006386339[2] |
| *Vitis vinifera* | WineGrape DORN1 | XP_002280641[2] |
| *Vitis vinifera* | WineGrape HMGB1/2/3 | XP_002280084[2] |
| *Vitis vinifera* | WineGrape MAPK3 | XP_002284807[2] |
| *Vitis vinifera* | WineGrape MAPK6 | F6HAX6[1] |
| *Vitis vinifera* | WineGrape PEPR1/2 | XP_002273607[2] |
| *Vitis vinifera* | WineGrape SERKL | D7STF6[1] |
| *Vitis vinifera* | WineGrape SR160 | XP_002278935[2] |
| *Vitis vinifera* | WineGrape WAKL | XP_002284700[2] |
| *Nicotiana tabacum* | Tobacco DORN1 | XP_016479299[2] |
| *Nicotiana tabacum* | Tobacco HMGB1/2/3 | XP_016457342[2] |
| *Nicotiana tabacum* | Tobacco HSYA | Q93WP8[1] |
| *Nicotiana tabacum* | Tobacco HSYB | Q93WP7[1] |
| *Nicotiana tabacum* | Tobacco MAPK3 | XP_016478177[2] |
| *Nicotiana tabacum* | Tobacco MAPK6 | O04362[1] |
| *Nicotiana tabacum* | Tobacco PEPR1/2 | XP_016436237[2] |
| *Nicotiana tabacum* | Tobacco SERKL | XP_016442040[2] |
| *Nicotiana tabacum* | Tobacco SR160 | NP_001312072[2] |
| *Nicotiana tabacum* | Tobacco WAKL | XP_016500142[2] |
| *Solanum lycopersicum* | Tomato SR160 | Q8GUQ5[1] |
| *Solanum lycopersicum* | Tomato DORN1 | XP_004246354[2] |
| *Solanum lycopersicum* | Tomato HMGB1/2/3 | XP_004232565[2] |
| *Solanum lycopersicum* | Tomato HSY | Q7XAD0[1] |
| *Solanum lycopersicum* | Tomato MAPK3 | Q84MI4[1] |
| *Solanum lycopersicum* | Tomato MAPK6 | NP_001234355[2] |
| *Solanum lycopersicum* | Tomato PEPR1/2 | XP_004235511[2] |
| *Solanum lycopersicum* | Tomato SERKL | NP_001234626[2] |
| *Solanum lycopersicum* | Tomato SYST | P27058[1] |
| *Solanum lycopersicum* | Tomato WAKL | K4DAS[1] |
| *Zea mays* | Maize DORN1 | NP_001146046[2] |
| *Zea mays* | Maize HMGB1/2/3 | XP_023157525[2] |
| *Zea mays* | Maize MAPK3 | NP_001140308[2] |
| *Zea mays* | Maize MAPK6 | NP_001152745[2] |
| *Zea mays* | Maize WAKL | XP_008671196[2] |
| *Zea mays* | Maize PEP1 | A0A0N9ZV66[1] |
| *Zea mays* | Maize PEPR1/2 | NP_001307735[2] |
| *Zea mays* | Maize SERKL | B4G007[1] |
| *Zea mays* | Maize SR160 | NP_001309780[2] |
| *Oryza sativa* | Rice DORN1 | XP_015635471[2] |
| *Oryza sativa* | Rice HMGB1/2/3 | XP_015651271[2] |

| | | |
|---|---|---|
| *Oryza sativa* | Rice MAPK3 | ABH01189[2] |
| *Oryza sativa* | Rice MAPK6 | Q84UI5[1] |
| *Oryza sativa* | Rice PEPR1/2 | XP_015648066[2] |
| *Oryza sativa* | Rice SERKL | Q67X31[1] |
| *Oryza sativa* | Rice SR160 | XP_015621030[2] |
| *Oryza sativa* | Rice WAKL | AAL34935[2] |
| *Hordeum vulgare* | Barley DORN1 | BAJ93092[2] |
| *Hordeum vulgare* | Barley HMGB1/2/3 | Q43481[1] |
| *Hordeum vulgare* | Barley MAPK3 | M0V3Q0[1] |
| *Hordeum vulgare* | Barley MAPK6 | BAJ90351[2] |
| *Hordeum vulgare* | Barley PEPR1/2 | BAK08322[2] |
| *Hordeum vulgare* | Barley SERKL | A2TLT1[1] |
| *Hordeum vulgare* | Barley SR160 | Q76CZ6[1] |
| *Hordeum vulgare* | Barley WAKL | BAJ97464[2] |
| *Homo sapiens* | Human HMGB1 | P09429[1] |
| *Homo sapiens* | Human MAPK3 | P27361[1] |
| *Homo sapiens* | Human MAPK6 | Q16659[1] |
| *Gallus gallus* | Chicken MAPK3 | NP_989481[2] |
| *Gallus gallus* | Chicken MAPK6 | Q5F3W3[1] |
| *Danio rerio* | Zebrafish MAPK3 | Q7ZVK8[1] |
| *Danio rerio* | Zebrafish MAPK6 | Q0H1F2[1] |

[1]UniProtKB (https://www.uniprot.org/uniprot/)

[2]NCBI GenBank (https://www.ncbi.nlm.nih.gov/genbank/)
